# Logistic LASSO Regression for Dietary Intakes and Breast Cancer

**DOI:** 10.3390/nu12092652

**Published:** 2020-08-31

**Authors:** Archana J. McEligot, Valerie Poynor, Rishabh Sharma, Anand Panangadan

**Affiliations:** 1 Department of Public Health, California State University, Fullerton, CA 92834, USA; 2 Department of Mathematics, California State University, Fullerton, CA 92834, USA; vpoynor@fullerton.edu; 3 Department of Computer Science, University of Houston, Houston, TX 77004, USA; rsharma26@uh.edu; 4 Department of Computer Science, California State University, Fullerton, CA 92834, USA; apanangadan@fullerton.edu

**Keywords:** diet, LASSO, breast cancer, NHANES

## Abstract

A multitude of dietary factors from dietary fat to macro and micronutrients intakes have been associated with breast cancer, yet data are still equivocal. Therefore, utilizing data from the large, multi-year, cross-sectional National Health and Nutrition Examination Survey (NHANES), we applied a novel, modern statistical shrinkage technique, logistic least absolute shrinkage and selection operator (LASSO) regression, to examine the association between dietary intakes in women, ≥50 years, with self-reported breast cancer (*n* = 286) compared with women without self-reported breast cancer (1144) from the 1999–2010 NHANES cycle. Logistic LASSO regression was used to examine the relationship between twenty-nine variables, including dietary variables from food, as well as well-established/known breast cancer risk factors, and to subsequently identify the most relevant variables associated with self-reported breast cancer. We observed that as the penalty factor (λ) increased in the logistic LASSO regression, well-established breast cancer risk factors, including age (β = 0.83) and parity (β = −0.05) remained in the model. For dietary macro and micronutrient intakes, only vitamin B12 (β = 0.07) was positively associated with self-reported breast cancer. Caffeine (β = −0.01) and alcohol (β = 0.03) use also continued to remain in the model. These data suggest that a diet high in vitamin B12, as well as alcohol use may be associated with self-reported breast cancer. Nonetheless, additional prospective studies should apply more recent statistical techniques to dietary data and cancer outcomes to replicate and confirm the present findings.

## 1. Introduction

In 2019, existing/prevalent cases of breast cancer in the United States reached more than 3.8 million, and approximately 42,000 women are expected to die from the disease in 2019 [1]. Although breast cancer incidence has been declining an average of 2.3% per year since 1990, 268,600 new cases of invasive breast cancer will be diagnosed in 2019, and breast cancer is still the second leading cause of cancer deaths for U.S. women. Nonetheless, it is only within the last 50 years that we have begun to investigate factors, including metabolic, dietary and other behavioral factors, that may be associated with breast cancer outcomes [2].

Modifiable risk factors such as dietary intakes, weight/obesity and physical activity, as well as alcohol consumption may independently influence a woman’s risk of breast cancer diagnoses [3]. Many epidemiologic studies have examined the relationship between dietary intakes and cancer risk/incidence. Overall, studies suggest that a largely plant-based diet, high in vegetables, whole fruits and fiber, and low in calories, as well as lower obesity have been shown to be protective against some types of cancers, particularly hormone-mediated cancers, including breast; but nonetheless, data are limited and inconclusive [3,4,5,6,7,8,9]. Further, due to the complexity and multitude of dietary variables, including whole dietary patterns of vegetable, fruit, fiber, fat, processed meats, sugary foods, lean meats, and a multitude of macro and micronutrients, most studies can and only have assessed either dietary patterns and/or single nutrients in relation to disease outcomes. Further, not only the volume of these modifiable factors, but also the multicollinearity between dietary macro and micronutrients may contribute to statistical challenges, specifically limiting the number and type of dietary variables included in the models, potentially resulting in conflicting findings and/or null results, and therefore warrant further investigation to elucidate the role of dietary intakes, both macro and micronutrients, with breast cancer.

The advent of big data science (BDs) has generated enormous amounts, varieties, and sources of complex data, and together with the availability of large open-source datasets and modern statistical techniques, has the vast potential for the creation of new knowledge, particularly in relation to primary and secondary disease prevention [10]. Beginning in 1999, the National Health and Nutrition Examination Survey (NHANES) began to continuously collect dietary, lifestyle, laboratory, physical examination, and other health information, amassing data on nearly 5000 participants per year on a multitude of health variables spanning over a decade [11]. Currently, NHANES is a large, open-source, publicly available dataset, which provides a unique opportunity to examine large, complex dietary, and other health information, including breast cancer diagnoses.

With the availability of vast amounts of diet and health data, and in conjunction with recent statistical techniques, we have an emerging opportunity to elucidate novel associations, patterns and clusters not previously observed with traditional statistical approaches and smaller datasets, potentially contributing to and providing a more robust understanding to the literature on the role of diet and breast cancer. One specific modern technique, the least absolute shrinkage and selection operator (LASSO) has garnered much attention [12]. Traditional regression techniques are limited in the analysis and synthesis of large numbers of covariates, including multicollinear variables, but to date, a majority of the data on diet and breast cancer outcomes have utilized traditional statistical techniques. LASSO is a regression-based methodology permitting for a large number of covariates in the model, and importantly has the unique feature penalizing the absolute value of a regression coefficient; thus, regulating the impact a coefficient may have on the overall regression. The greater the penalization, the greater the shrinkage of coefficients, with some reaching 0, thus automatically removing unnecessary/uninfluential covariates [13,14,15].

Therefore, for the present study, we aimed to investigate via modern statistical techniques, specifically LASSO regression, the relationship between dietary intakes, obesity, and other risk factors on self-reported breast cancer. To our knowledge and in reviewing the diet and cancer literature, this is the first study to apply LASSO regression techniques to dietary intakes and breast cancer.

## 2. Materials and Methods

### 2.1. Study Design

Beginning in 1999, NHANES transitioned to a continuous ongoing cross-sectional survey conducted by the National Center for Health Statistics at the Centers for Disease Control and Prevention (CDC). The CDC, via NHANES, collects data on the health and nutritional status of noninstitutionalized U.S. adults and children using probabilistic, multistage sampling and oversampling to achieve a nationally representative sample of the U.S. population. The interview component of NHANES ascertains information on demographic, socioeconomic, and health-related factors and includes a 24-h dietary recall assessment. Detailed information on survey design and methodology have been previously published [11].

### 2.2. Sample

**Figure 1:**
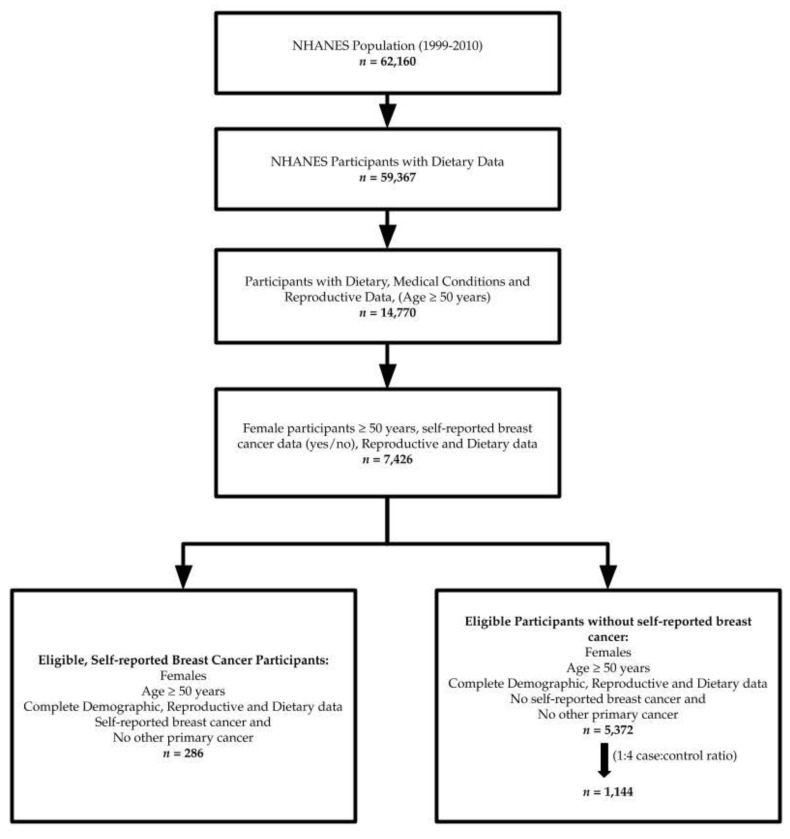
Flowchart of study participants.

The 1999–2010 cycle of NHANES included a nationally representative population of 62,160 with 59,367 participants with dietary data. We limited our analyses to adults ≥50 years (*n* = 14,770) who had demographic data, participated in dietary assessment and had medical conditions and reproductive data (*n* = 14,770), and then further honed our analyses to female participants, ≥50 years with non-missing data on primary breast cancer diagnoses, reproductive, and dietary data (*n* = 7426). Our final study sample of women with breast cancer included women ≥50 years with self-reported breast cancer and no other cancers, as well as complete/non-missing demographic, reproductive, and dietary data (*n* = 286); per the NHANES definition. This required that all relevant variables associated with the 24-h dietary recall are non-missing and have a value. Our initial sample of women without breast cancer included women ≥50 years with no self-reported breast cancer and no other cancers, as well as complete/non-missing demographic, reproductive, and dietary data (*n* = 5372). In addition, of the women without self-reported breast cancer for the present analyses (*n* = 5372), we further random-sampled for optimal statistical power, maintaining an approximate optimal ratio of up to 1:4 for cases [16], resulting in a final study sample of women with self-reported breast cancer (*n* = 286) and women without self-reported breast cancer (*n* = 1144) (Figure 1). The CDC Institutional Review Board approved NHANES and all participants provided written informed consent. The study protocol review was conducted and approved by the Internal Review Board (IRB) of the California State University, Fullerton (HSR# 18-19-250).

### 2.3. Breast Cancer Data

In the “Medical Conditions” portion of the NHANES interview, health conditions and medical history were collected on adults, including cancer malignancies. Participants were queried on the following: “Have you ever been told by a doctor or other health professional that you had cancer or a malignancy of any kind?” And then followed by, “What kind of cancer was it?” Age at diagnoses was also collected via the following question: “How old were you when breast cancer was first diagnosed?” Women reporting only breast cancer diagnoses were included in the present study.

### 2.4. Dietary Intake

Dietary macro- and micronutrient intakes, from food only, were obtained from the total nutrient intakes data set. Dietary intakes were reported via a 24-h dietary recall in which respondents reported individual foods (and drinks) consumed during the midnight-to-midnight 24-h period prior to the in-person dietary interview. Coding of interview data and conversion to total nutrient intakes were done by NHANES using the USDA Food and Nutrient Database for Dietary Studies, 5.0 (FNDDS 5.0) [17]. The FNDDS 5.0 nutrient values were based on the USDA National Nutrient Database for Standard Reference, release 24 [18].

### 2.5. Other Measures

Age and race/ethnicity were obtained from the demographic variables and sample weights data set. NHANES categorizes race/ethnicity into four groups: non-Hispanic white, Hispanic, African American and other/multi-racial. Body mass index [BMI (kg/m^2^)] was obtained from the body measures data set. Parity was ascertained via the following NHANES question, “How many times have you been pregnant? (Again, be sure to count all your pregnancies including (current pregnancy), live births, miscarriages, stillbirths, tubal pregnancies or abortions)”. Alcohol and caffeine consumption were expressed as grams and mg per day, respectively, and obtained via 24-h dietary recall data.

### 2.6. Statistical Analysis

Statistical analyses were conducted in R Statistical Software (version 3.5.2). The “survey” package (version 4.0) in R was utilized for the univariate analyses accounting for the stratified, multistage probability cluster sampling design in NHANES. NHANES provides sampling weights for analytical purposes, which account for oversampling of certain subgroups, differences between the sample and the population due to nonresponse, and population sizes. More specifically, NHANES provides sampling weights to be used for dietary analyses, which also account for the fact that not all participants completed the dietary interview and that different days of the week were represented in the 24-h periods for which dietary intake was assessed. Our study sample consisted of 6 cycles of continuous NHANES data from 1999–2010, thus dietary weights were adjusted to reflect the U.S. population. Due to different reference populations, the 4-year dietary weights for 1999–2002 were adjusted, while the remaining cycles for 2003–2010 adjusted the 2-year dietary weights. The NHANES stratification variable (SDMVSTRA) and primary sampling unit variable (SDMVPSU) were incorporated according to the survey design to appropriately adjust the variance estimates.

Descriptive and dietary variables were tested for normality and were log-transformed as appropriate, including all dietary variables. Specifically, statistical analyses were conducted on log transformed variables, including univariate tests and LASSO regression; however, mean data and 95% confidence intervals shown are on the raw/non-log transformed data. Height and weight data were used to calculate BMI (kg/m^2^). Univariate analyses, specifically *t*-test for continuous variables and chi-square for discrete variables, respectively, were performed for demographic data including age and ethnicity, as well as age at first menarche, parity, and BMI. Also, mean intakes for macro- and micronutrients (from food only) were calculated and t-test analyses, as well as respective 95% confidence intervals of the mean differences were conducted to examine differences in dietary nutrient intakes between women with and without self-reported reported breast cancer diagnoses. All statistical tests were two-sided with 0.05 significance levels.

The logistic LASSO model is a shrinkage method that can actively select from a large and potentially multicollinear set of variables in the regression, resulting in a more relevant and interpretable set of predictors [12]. LASSO performs via a continuous shrinking operation, minimizing regression coefficients in order to reduce the likelihood of overfitting, however, the technique is computed so as to shrink the sum of the absolute value of regression coefficients, forcing and producing coefficients that are exactly 0, thus selecting for the nonzero variables to remain in the model.

**Figure 2:**
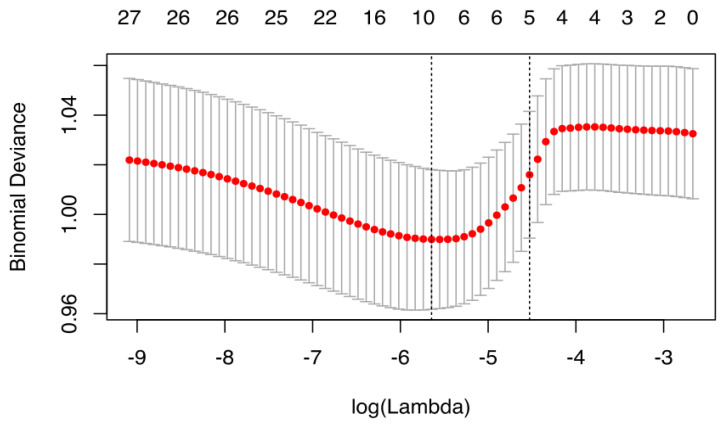
Cross validation plot for the penalty term.

We utilized the “glmnet” package (version 2.0-16) to fit the logistic LASSO regression. The dietary weights were normalized and incorporated in the same fashion as standard weighted regression [19]. The covariates were not standardized as this would cause the weighting structure to be lost as described previously [20]. But, briefly, McConville, 2011 [19] showed that for survey-weighted LASSO regression analysis, the covariates should not be standardized as the inverse inclusion weights associated with each participant would be lost. The inverse inclusion weights are the normalized sampling weights. Furthermore, our analysis was performed on the log scale of the covariates, which minimizes the range of the covariate values, thus no one covariate dominated in the model due to a larger/wider range. We utilized ten-fold cross-validation to select the penalty term, λ. The binomial deviance was computed for the test data as measures of the predictive performance of the fitted models. The built-in function in R produces two automatic λ’s—one that minimizes the binomial deviance and one representing largest λ that is still within 1 standard error of the minimum binomial deviance. We opted for the latter λ as it results in stricter penalty allowing us to reduce the number of covariates even further than the former λ. For the present analyses, the λ values ranged from 0.00009 to 0.06937 with a minimal binomial deviance achieved at 0.0035 and more stringent value of 0.0108 (Figure 2). The standard errors of the LASSO coefficients were obtained via bootstrapping within the primary sampling unit and strata [21].

For the logistic LASSO regression, self-reported breast cancer was included as the dependent variable, Y, and coded as 0 for no cancer and 1 for presence of breast cancer. Additionally, we included all 21 dietary variables from food, in addition to alcohol and caffeine consumption, available during the respective years of analyses, via the NHANES 24-h dietary recall data, including: energy (Kcal), % energy from carbohydrate, % energy from fat, % energy from protein, % energy from fat, cholesterol (mg), fiber (g), folate (μg), vitamin B12 (μg), vitamin B6 (mg), thiamin (vitamin B1, mg), riboflavin (vitamin B2, mg), calcium (mg), phosphorous (mg), magnesium (mg), iron (mg), vitamin A (RE), vitamin C (mg), vitamin E (mg), zinc (mg), sodium (mg), potassium (mg), caffeine (mg), and alcohol (g). Energy dense macronutrients, including dietary fat, carbohydrate, and protein were adjusted for energy intakes and included in the logistic LASSO regression model as % energy of the respective macronutrient. All dietary variables were included as continuous variables in the model. All variables evaluated as potential confounders and specifically those shown to be previously associated with breast cancer risk (based on literature) were also included in the model: age (continuous), age at menarche (continuous), and parity (continuous). We also included BMI in our model, however BMI data were available only on a subset of participants, *n* = 279 (with self-reported breast cancer) and *n* = 1116 (without breast cancer), and analyses for these data were conducted on the respective sample size. We also examined time since breast cancer diagnoses (age at interview minus age at diagnoses), and conducted correlation analyses between the time since diagnoses variable and dietary intakes. Specifically, independent pairwise correlation tests were performed on the log scale of all dietary variables with time since diagnoses.

## 3. Results

Demographic and well-established breast cancer risk factor data (including respective % (±SD) or mean (± SD)) are shown in Table 1. Significant differences (*p* ≤ 0.05) between women with self-reported breast cancer and women without were observed for age 68.46 (0.74) vs. 63.19 (0.36) years, age at first menarche (12.62 (0.13) vs. 12.89 (0.06) years), and ethnicity, where women with self-reported breast cancer were more likely to be older, had a younger age at menarche, were less parous, and were more likely to be to be non-Hispanic white compared with women without self-reported breast cancer (88% vs. 77%, respectively). Correlation analyses with dietary intakes and time since diagnoses showed no correlation between variables, except for alcohol (r = 0.797, *p* = 0.013) (data not shown).

Table 2 presents data on dietary intakes between women with and without self-reported breast cancer. Univariate analyses suggest a statistically significant borderline higher intake of dietary vitamin B12 (μg/d) in women with self-reported breast cancer compared with women without self-reported breast cancer ((5.02 (0.75) vs. 4.17 (0.15), respectively; 95% CI: (−0.632, 2.34); *p* = 0.08)). Women with self-reported breast cancer also had higher alcohol (g) consumption ((5.31 (1.01) vs. 3.17 (0.49)) as well as vitamin A (IU) intakes ((685.55 (75.15), 648.52 (18.85)), however these variables did not reach statistical significance (*p* = 0.19).

Table 3 shows data adjusted for all the dietary variables, including macro- and micronutrient intakes, as well as well-established variables associated with breast cancer. The logistic LASSO regression results showed that of the well-established breast cancer risk factors, age (β = 0.83) and parity (β = −0.05) contributed to self-reported breast cancer. Specifically, age was positively associated with breast cancer, while parity was inversely associated. For dietary macro- and micronutrient intakes, only vitamin B12 (β = 0.07) was positively associated with self-reported breast cancer. Alcohol (β = 0.03) use also continued to remain in the model and was positively associated, while caffeine was inversely (β = −0.01) related to self-reported breast cancer diagnoses.

**Figure 3:**
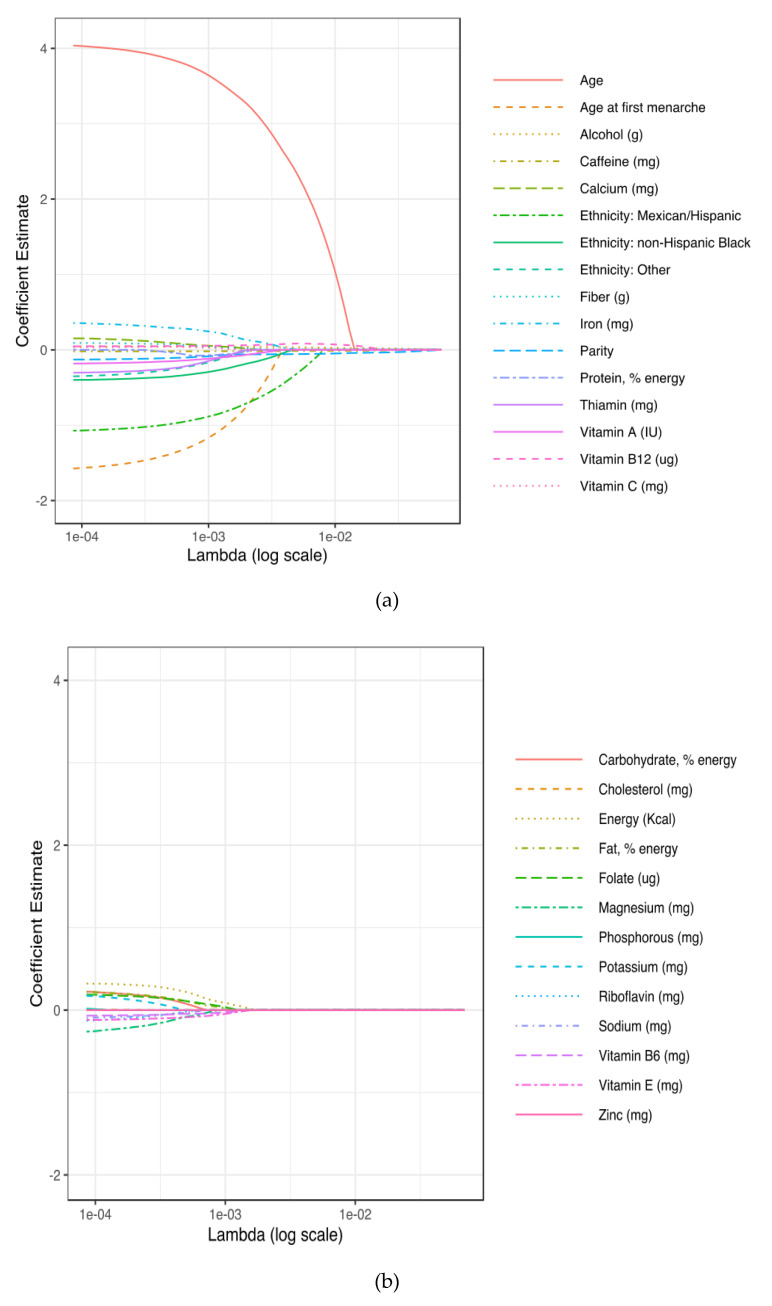
Plots for LASSO regression coefficients over different values of the penalty parameter. In (**a**), data shown are the sixteen variables that remained in the model the longest as the penalty term increased; in (**b**), data shown are the remaining variables in the model.

Figure 3a,b shows results on the 29 variables included in the LASSO regression and their corresponding coefficients for the different values of the penalty parameter. We observed that at λ = 0.00009, all 29 variables remain in the model (i.e., are nonzero). In Figure 3a, we show that 16 variables (age, age at first menarche, alcohol, caffeine, calcium, ethnicity, fiber, iron, parity, protein, thiamin, vitamin A, vitamin B12, and vitamin C) remained longest in the model as the penalty term increased with the other remaining variables approaching zero more quickly (Figure 3b). As λ increases to 0.01079, only five variables, potentially the most influential on self-reported breast cancer, remain in the model. Specifically, as λ approaches 0.01079, age, vitamin B12, caffeine, alcohol, and parity confer the largest signal in the model.

## 4. Discussion

Using a large, cross-sectional, nationally representative sample, in conjunction with modern robust statistical techniques, we applied logistic LASSO regression, which minimizes multicollinearity between dietary variables, to assess the relationship between dietary intakes and breast cancer diagnoses. Via LASSO, we also accounted for well-established breast cancer risk factors, while simultaneously selecting for relevant coefficients from a multitude of variables, ultimately removing all other unrelated variables. Our initial univariate analyses showed that age, age at first menarche, ethnicity, and also vitamin B12 from food, was related to breast cancer. In the ultimate logistic LASSO regression, well-established breast cancer risk factors, including older age and lower parity were associated with increased breast cancer, and vitamin B12, and alcohol and caffeine intakes were also related to self-reported breast cancer. Thus, we showed that increased alcohol consumption and reduced caffeine use were associated with an increase in breast cancer, with only vitamin B12 from diet remaining in the model. To the knowledge of the authors, this is the first study to utilize the powerful LASSO shrinkage technique to assess the relationship between the multitude of dietary variables and other risk factors with breast cancer diagnoses.

Our results of the association of well-established breast cancer risk factors, including age, race/ethnicity, age at menarche, and parity have been observed previously [22,23,24,25,26,27]. Age continued to remain in the model and was strongly related to breast cancer. In previous studies, aging has been clearly related to breast cancer diagnoses with a majority occurring in postmenopausal women, and more than 77% occurring for women 50 years of age or older [23,28]. Similar to our results, well-established reproductive factors, due to endogenous estrogen exposure, including early age at first menarche (<11 years), pregnancy (ever pregnant), and number of children have been shown to be linked to breast cancer risk [25,26,27,28].

Alcohol use has been consistently shown to increase breast cancer risk [3,29,30,31,32]. The International Agency for Research on Cancer (IARC) has designated alcohol as a carcinogenic risk factor [29], with the association being observed in both pre- and postmenopausal women. Earlier case/control studies showed increased risk with one reporting a 90% increase in breast cancer risk (OR: 1.9; 95% confidence interval, CI, 1.5–2.4) in ever drinkers compared with never drinkers [31,32], with subsequent epidemiologic studies establishing a positive association between increased quantity of alcohol consumption, showing a dose-response and causal relationship [29,33,34,35,36]. Our results are consistent with these previous findings, showing that even after accounting for a multitude of dietary factors, alcohol remained positively associated with breast cancer diagnoses. However, no previous studies have utilized statistical shrinkage techniques to assess the relationship between alcohol use and breast cancer.

Previous studies have shown that caffeine and/or coffee consumption may be associated with reduced breast cancer risk, but data remain equivocal [37,38,39,40,41,42]. There are several plausible mechanisms by which caffeine and/or coffee consumption may influence breast cancer risk, including the role of caffeine in estrogen metabolism, antioxidant actions of coffee/tea, and tumor differentiation and DNA methylation [43,44,45,46,47]. A recent study of 335,060 women participating in the European Prospective Investigation into Nutrition and Cancer (EPIC) Study reported that caffeinated coffee intake was associated with lower risk of postmenopausal breast cancer: adjusted HR = 0.90, 95% confidence interval (CI): 0.82 to 0.98, for high versus low consumption; *p*_trend_ = 0.029 [40]. Another large, longitudinal cohort study, the Nurses’ Health Study, also showed a significant, yet weak, inverse association of caffeine intake with postmenopausal breast cancer for the highest quintile of intake compared to the lowest, RR: 0.88 (95% CI = 0.79 to 0.97, *p*_trend_ = 0.03) [43]. However, other prospective cohort studies have shown little to no association between caffeinated coffee intake and risk of breast cancer [41,42,48,49]. It is also plausible that other constituents in coffee and/or tea may either interact with caffeine and/or serve as a proxy in conferring protection against breast cancer [50,51,52,53], however our findings are consistent with the larger cohort studies in suggesting an inverse relationship between caffeine intake and breast cancer.

Our findings of a positive relationship between vitamin B12 and breast cancer diagnoses have been reported in previous studies [54,55,56,57]. However, other studies have found an inverse and/or no association [58,59]. In a prospective study of 936 incident breast cancer cases, dietary vitamin B12 was associated with increased risk of breast cancer (HR: Quartile 4 vs. Quartile 1 = 1.21 (1.00, 1.46); *p*_trend_ = 0.06) [55]. Recent EPIC cohort study investigations suggest a weak positive association between plasma vitamin B12 and breast cancer risk, however the association was attenuated by alcohol and/or folate status [54]. As a plausible mechanism, several water-soluble vitamins, including folate, vitamin B6, and vitamin B12 play a critical role in one-carbon metabolism, generating substrates for DNA methylation and DNA syntheses, and therefore modulate cancer risk [60,61,62,63]. Vitamin B12, primarily found in meat and dairy products, is involved in DNA methylation and may interfere with gene expression and function, whereby potentially conferring neoplastic cell growth [64,65]. Comparison of our findings with previous studies confirms a positive association of vitamin B12 with breast cancer, however data are inconclusive due to alcohol, folate, and/or epigenetic interactions and should be studied further.

Strengths of our study include the large sample size available via NHANES, which provides sufficient power to detect clinically relevant differences, and the generalizability of results due to the nationally-representativeness of the NHANES survey data. Additionally, another strength is representation via oversampling of diverse racial/ethnic subgroups, such as Hispanics and African Americans, inherent in the NHANES survey methodology, enabling inclusion of groups that are often underrepresented in the scientific literature on diet and breast cancer. Limitations include the retrospective, cross-sectional design, which does not allow for causal inference, and self-reported data on diet and breast cancer. Further, it is feasible that women may have changed their diet post-diagnoses, which may influence findings, nonetheless, our correlation analyses of time since diagnoses found only one association, possibly due to multiple comparisons, between dietary intakes and time since diagnoses in women with self-reported breast cancer, potentially suggesting little to no change in diet from time since diagnoses. We also reported on dietary intakes from food only, and not on supplemental intake. Supplement data availability during the study time period were limited, and therefore only dietary data were examined in order to increase sample size and preserve power to detect associations.

## 5. Conclusions

In conclusion, to the knowledge of the authors, we showed for the first time, via a powerful shrinkage technique, that LASSO regression can be a viable option to narrow and decipher the role of a multitude of dietary factors and their relation to breast cancer diagnoses. We showed that established breast cancer factors, including age and parity continue to be associated with breast cancer diagnoses, and that alcohol use was positively associated, while caffeine intake was inversely related to breast cancer diagnoses. Our shrinkage analyses findings also suggest a potential role of dietary vitamin B12 intake and breast cancer diagnoses, however LASSO applications and use in assessing dietary intakes and breast cancer need to be confirmed in other prospective studies and warrant further investigation.

## Figures and Tables

**Table 1:** Descriptive and other characteristics in participants with and without self-reported breast cancer.

Descriptive Variable	Women with Self-Reported Breast Cancer (*n* = 286)	Women without Self-Reported Breast Cancer (*n* = 1144)	*p*-Value
Mean age (± SD)	68.46, (0.74)	63.19, (0.36)	<0.001
Parity, mean (± SD)	2.49, (0.17)	2.70, (0.07)	0.15
Age at first menarche, mean (± SD)	12.62, (0.13)	12.89, (0.06)	0.06
Ethnicity, *n*, (%)			
Non-Hispanic White	203, (88%)	595, (77%)	<0.001
Non-Hispanic Black	44, (7.2%)	219, (10.9%)	
Hispanic	32, (2.6%)	294, (8.1%)	
Unknown/Other	5, (2.3%)	36, (4.5%)	
BMI (kg/m^2^) ^1^, mean (± SD)	28.89, (0.55)	29.38, (0.33)	0.43

^1^ BMI variable, *n* = 279 with breast cancer, *n* = 1116 without breast cancer.

**Table 2:** Dietary macro- and micronutrient intakes in women with and without self-reported breast cancer.

Descriptive Variable Mean (SD) ^1^	Women with Self-Reported Breast Cancer (*n* = 286)	Women without Self-Reported Breast Cancer (*n* = 1144)	95% CI (Difference of Means) ^1^	*p*-Value
Energy (Kcal)	1638 (43.60)	1648 (28.23)	(−109.59, 91.01)	0.46
Carbohydrate (g)	205.38 (6.46)	204.75 (3.77)	(−14.34, 15.57)	0.36
Carbohydrate, % energy	50.43 (0.86)	50.44 (0.48)	(−2.00, 1.98)	0.80
Protein (g)	64.20 (2.38)	65.45 (1.31)	(−6.46, 3.97)	0.68
Protein, % energy	15.89 (0.30)	16.10 (0.18)	(−0.944, 0.520)	0.72
Total Fat (g)	61.86 (2.05)	63.99 (1.47)	(−6.83, 2.64)	0.50
Fat, % energy	33.57 (0.60)	33.97 (0.39)	(−1.88, 1.06)	0.78
Cholesterol (mg)	213.80 (10.56)	226.50 (7.70)	(−40.46, 15.05)	0.40
Fiber (g)	15.23 (0.72)	14.85 (0.34)	(−1.25, 2.02)	0.38
Folate (μg)	353.49 (13.81)	347.07 (8.40)	(−26.49, 39.34)	0.38
Vitamin B12 (μg)	5.02 (0.75)	4.17 (0.15)	(−0.632, 2.34)	0.08
Vitamin B6 (mg)	1.61 (0.07)	1.60 (0.04)	(−0.16, 0.19)	0.45
Thiamin (mg)	1.36 (0.07)	1.39 (0.04)	(−0.18, 0.15)	0.73
Riboflavin (mg)	1.89 (0.07)	1.88 (0.04)	(−0.164, 0.16)	0.45
Calcium (mg)	772.74 (27.24)	780.44 (20.44)	(−67.42, 52.03)	0.21
Phosphorous (mg)	1082 (35.87)	1096 (20.13)	(−91.45, 63.47)	0.49
Magnesium (mg)	253.27 (9.13)	256.33 (4.79)	(−24.00, 17.89)	0.65
Iron (mg)	13.24 (0.56)	12.84 (0.29)	(−0.93, 1.72)	0.25
Vitamin A (IU)	685.55 (75.15)	648.52 (18.85)	(−116.40, 190.45)	0.19
Vitamin C (mg)	87.68 (4.14)	92.04 (4.81)	(−16.66, 7.94)	0.28
Vitamin E (mg)	6.66 (0.40)	6.67 (0.19)	(−0.93, 0.91)	0.52
Zinc (mg)	9.76 (0.35)	9.61 (0.24)	(−0.61, 0.92)	0.24
Sodium (mg)	2665 (84.90)	2768 (59.30)	(−289.68, 81.87)	0.80
Potassium (mg)	2452 (61.65)	2476 (39.86)	(−158.56, 109.05)	0.38
Caffeine (mg)	154.56 (14.42)	174.94 (11.62)	(−57.97, 17.21)	0.38
Alcohol (g)	5.31 (1.01)	3.17 (0.49)	(0.08, 4.21) ^2^	0.19

^1^ Mean (SD) macro- and micronutrient, and 95% CI data shown are on the raw data; *p*-value data shown are on log transformed data; ^2^ Log transformed 95% CI = (−0.20, 1.03).

**Table 3:** The estimated coefficients for logistic least absolute shrinkage and selection operator (LASSO) regression between dietary data, and well-established breast cancer risk factors with self-reported breast cancer.

Variables	Coefficients (Bootstrap SE)
Well-established Variables	
Age (years)	0.83 (0.41)
Parity (# live births)	−0.05 (0.03)
Age at first menstrual cycle	0
Alcohol (g)	0.03 (0.02)
Other Variables	
Caffeine (mg)	−0.01 (0.02)
Mexican/Hispanic	0
Non-Hispanic Black	0
Other	0
Dietary Variables	
Energy (Kcal)	0
Carbohydrate, % energy	0
Protein, % energy	0
Fat, % energy	0
Cholesterol (mg)	0
Fiber (g)	0
Folate (μg)	0
Vitamin B12 (μg)	0.07 (0.05)
Vitamin B6 (mg)	0
Thiamin (Vitamin B1) (mg)	0
Riboflavin (Vitamin B2) (mg)	0
Calcium (mg)	0
Phosphorous (mg)	0
Magnesium (mg)	0
Iron (mg)	0
Vitamin A (RE)	0
Vitamin C (mg)	0
Vitamin E (mg)	0
Zinc (mg)	0
Sodium (mg)	0
Potassium (mg)	0
